# Early elevation of BACE1 in dementia

**DOI:** 10.18632/aging.203727

**Published:** 2021-11-29

**Authors:** Carlo Cervellati, Giuseppe Valacchi, Giovanni Zuliani

**Affiliations:** 1 Department of Translational Medicine and for Romagna, University of Ferrara, Ferrara 44121, Italy; 2 Dipartimento dell'Ambiente e della Prevenzione, Universita' di Ferrara, Ferrara 4644121, Italy; 3 Animal Science Department, Plants for Human Health Institute, NC State University, Kannapolis, NC 28081, USA; 4 Department of Food and Nutrition, Kyung Hee University, Seoul, South Korea

**Keywords:** Alzheimer’s disease, amyloid- β, BACE1, mild cognitive impairment

Interest in the role of Beta-secretase1 (BACE1) in Alzheimer’s disease (AD) pathogenesis and pathophysiology has been remarkably growing in the last 10 years [1]. This transmembrane aspartyl protease catalyzes the rate-limiting step of the sequential cleavage of amyloid precursor protein (AβPP) to form Aβ fragments which will then aggregate in so-called senile plaques [1].

The burst of published literature on the topic mainly stems from the potential significance of this key-enzyme of amyloidogenic pathway as pharmacological target [2]. Based on our published research we have suggested another possible clinical application of BACE1. Indeed, inspired by mounting evidence of BACE1 increase in brain, CSF, and plasma, in AD patients compared to healthy controls [1], we have started to evaluate the diagnostic performance of serum activity of the enzyme. Taking a stock of our results, from the analysis of total 817 subjects, we have found that serum BACE1 activity is around 30% higher in patients with AD, vascular dementia (VD), mixed dementia (AD & VD) compared to controls [3,4]. Then, we moved the lens to amnestic and non-amnestic mild cognitive impairment (a- and na-MCI, respectively), with the former being more prone to convert into AD compared to the latter [5]. This recent investigation has revealed that serum BACE1 activity: 1) is similarly higher (around 30%) in both aMCI and naMCIs as compared to healthy controls; 2) is higher in aMCI converting to dementia compared to stable MCI when considering patients with a less compromised cognitive status; 3) is not a predictor of conversion from aMCI to dementia.

The above findings can be discussed as follow:

1. The increase in BACE1 serum activity in MCI patients suggests that this enzyme is more active before the onset of overt dementia. Moreover, this abnormality appears to occur irrespectively of the possible clinical course of these patients (since it is observed in na- and a-MCI). Previous post-mortem and CSF findings on MCI indicate that the precocious increase in peripheral BACE1 might reflect CNS conditions [1]. More intriguingly, a recent longitudinal study has shown that plasma BACE1 level is associated with progressive axonal damage/neurodegeneration in AD critical brain region, already in cognitively and physically healthy individuals (at risk of AD) [6]. Consistently, we have also observed an association between high BACE1 and the presence of brain atrophy in aMCI [5].

These findings are not surprising, since it is well-known that Aβ deposition may precede AD clinical symptoms by approximately 15-20 years. It is also well-established that synapse loss and dysfunction is a key feature in many neurodegenerative diseases including dementia and, in particular, AD [1,6]. BACE1 has the potential to be an active player in these detrimental changes, since it is able to shed several substrates, besides AβPP, implicated in, myelinization, synaptic plasticity and axonal organization. Indeed, recent approaches to identify novel BACE1 substrates, have uncovered 19% of the 183 neuronal membrane proteins, with most of them involved in neurite outgrowth and synapsis formation, requiring this enzyme for their shedding [1,6]. It is thus conceivable that, undesirable BACE1 translational activation could occur precociously, and this could prime subclinical neurodegeneration.

2. The selective increase of serum BACE1 activity in converter aMCI with a higher MMSE might suggest that this could be a risk factor for dementia in those patients where the cognitive decline has not overtly taken place. It is tempting to speculate, that the contribution of BACE1 in the clinical trajectory to dementia is tangible only when other pathogenic players have not yet become predominant. Indeed, while it is widely recognized that Aβ formation is the initial event in AD, it has also become increasingly apparent that secondary abnormalities such as tau and neuroinflammation might play a major role in the subsequent neurodegeneration [7]. In other words, for MCIs with pronounced cognitive symptoms, the impact of tauopathy and inflammation may be dominant and mask the contribution of BACE1 in the progression to the disease. Apparently, the results we obtained by Cox model (median follow up of 32 months) do not support the use of serum BACE1 activity as prediction biomarker for AD and dementia in general [5]. However, it must be underscored that our final sample was definitely underpowered for this kind of analysis. Given the above mentioned results and the clear trend of Cox curve, we believe that a larger number of MCI with good cognitive performance could give different results

In summary, BACE1 up-regulation might be one of the first abnormal event in AD (and VD). Within this long preclinical phase of the disease, which lasts years before the diagnosis of MCI state, BACE1 may be the key-player of the initial, and still asymptomatic, neuropathological changes. In this time frame, the evaluation of its serum activity might have clinical relevance as early biomarker, mostly for screening subjects to enroll in clinical trials. The time when Aβ accumulation is the prominent alteration, and the specific weight of other pathogenic actors is still relatively low, BACE1 could also be candidate therapeutical target. As recently suggested by Panza et al. [2], large random clinical trials with BACE1 inhibitors have failed (for lack of benefit or the occurrence of adverse effects) most likely because they were administered on patients experiencing the irreversible phase of the disease (mild-to-moderate AD). Indeed, in these treated patients the significant decrease in cerebrospinal fluid Aβ (but not in amyloid-PET), is not accompanied by cognitive benefit [2]. This could mean that BACE1-mediated formation of Aβ has lost its “primacy”, and other pathogenic players could have taken over the lead.
